# T94. HS-CRP TO EVALUATE CHRONIC INFLAMMATION AND CARDIOVASCULAR DISEASES IN SCHIZOPHRENIA

**DOI:** 10.1093/schbul/sby016.370

**Published:** 2018-04-01

**Authors:** Rahma Nefzi, Amine Larnaout, Ben Ammar Hanen, Emira Khelifa, Aissa Amina, El Hechmi Zouhaier, Guemira Fethi

**Affiliations:** 1 Razi Hospital; 2 Salah Azaiz Institut

## Abstract

**Background:**

The metabolic syndrome is a combination of risk factors for cardiovascular disease (RFCV). These complications are responsible for a significant excess mortality found in patients with schizophrenia. C-reactive protein (CRP), the main protein of the acute phase of the inflammatory process, has been chosen as one of the most informative biomarkers for predicting vascular death and major cardiovascular events at 10 years of age. It is the moderate and chronic increase in CRP levels measured by high-sensitivity C – reactive protein (hs-CRP) that represents a risk factor for cardiovascular disease. In the meanwhile, the results of research on autoimmunity and inflammation during psychosis described high levels of inflammatory markers in schizophrenia. In fact, chronic inflammation, measured by high blood C-reactive protein level, has been described in schizophrenia.

The aim of this work was to evaluate the association between serum levels of high-sensitivity C – reactive protein, as a marker of chronic inflammation, metabolic syndrome and cardiovascular risk in a cohort of Tunisian patients with schizophrenia during remission.

**Methods:**

A cross-sectional and retrospective descriptive study was conducted at the “F” psychiatry department at the Razi Hospital, including 80 patients with schizophrenia in period of clinical remission. The evaluation focused on 11 cardiovascular risk factors: age, family history of early heart disease, physical inactivity, alcohol consumption, smoking, type 2 diabetes, android obesity, the elevation of total cholesterol, the decrease of hdl-cholesterol, high blood pressure, elevation of triglycerides. A dosage of high-sensitivity C – reactive protein was performed.

**Results:**

25 patients (31%) met the criteria for metabolic syndrome of the International Diabetes Federation (2006). 13 patients (16%) had none of the 5 diagnostic criteria for metabolic syndrome. The average number of cardiovascular risks was 3.66.

22% of patients had significant cardiovascular risk (number of risk factors ≥ 5).

The average measured CRP us was 3.43 ± 2.08 mg / l. Taking only the measure of hs-CRP as RFCV, 64% of our patients had a moderate cardiovascular risk and 38% had a significant risk.

Hs-CRP levels were not associated with metabolic syndrome (p=0.4). However, a strong association was found between high levels of hs-CRP and high risks for cardiovascular disease (p=0.006).

**Discussion:**

Chronic inflammation plays a role in the pathophysiology of many chronic diseases, including cardiovascular diseases. It also plays an important role in the pathogenesis of schizophrenia. The role of the immuno-inflammatory system in schizophrenia arouses interest in immuno-psychiatric research.

The association between chronic inflammation and cardiovascular diseases in schizophrenia could lead to treatments that would prevent the progression of both diseases overall.

